# Plasma CXCL14 as a Candidate Biomarker for the Diagnosis of Lung Cancer

**DOI:** 10.3389/fonc.2022.833866

**Published:** 2022-06-08

**Authors:** Peng-Fei Tian, Yu-Chen Ma, Dong-Sheng Yue, Fan Liang, Chen-Guang Li, Chen Chen, Hua Zhang, Xiao-Yan Sun, Wu-Hao Huang, Zhen-Fa Zhang, Guang-Biao Zhou, Gui-Zhen Wang, Bin Zhang, Chang-Li Wang

**Affiliations:** ^1^ Department of Lung Cancer, Tianjin Lung Cancer Center, National Clinical Research Center for Cancer, Key Laboratory of Cancer Prevention and Therapy, Tianjin’s Clinical Research Center for Cancer, Tianjin Medical University Cancer Institute and Hospital, Tianjin, China; ^2^ Clinical Medical Research Center, Xianyang Central Hospital, Xianyang, China; ^3^ State Key Laboratory of Molecular Oncology, National Cancer Center/National Clinical Research Center for Cancer/Cancer Hospital, Chinese Academy of Medical Sciences and Peking Union Medical College, Beijing, China

**Keywords:** lung cancer, CXCL14, biomarker, diagnosis, early detection

## Abstract

**Background:**

Effective biomarkers for early diagnosis of lung cancer are needed. Previous studies have indicated positive associations between abnormal circulating cytokines and the etiology of lung cancer.

**Methods:**

Blood samples were obtained from 286 patients with pretreatment lung cancer and 80 healthy volunteers. Circulating cytokine levels were detected with a Luminex assay and enzyme-linked immunosorbent assay (ELISA). Urine samples were obtained from 284 patients and 122 healthy volunteers. CXC chemokine ligand 14 (CXCL14) expression in tumors and nontumor regions of lung tissues from 133 lung cancer cases was detected by immunohistochemical (IHC) staining and immunofluorescence (IF) staining of formalin fixed paraffin-embedded (FFPE) tissues.

**Results:**

Compared with healthy volunteers, a 65.7-fold increase was observed in the level of CXCL14 in the plasma of lung cancer patients, and a 1.7-fold increase was observed in the level of CXCL14 in the urine of lung cancer patients, achieving a 0.9464 AUC (area under the curve) value and a 0.6476 AUC value for differentiating between lung cancer patients and healthy volunteers, respectively. Stromal CXCL14 expression was significantly associated with advanced pathologic stage (*P*<0.001), pathologic N stage (*P*<0.001), and recurrence and metastasis (*P*=0.014). Moreover, multivariate analysis suggested stromal CXCL14 expression as an independent predictor of DFS and OS.

**Conclusions:**

Our study demonstrates that CXCL14 might serve as a potential diagnostic and prognostic biomarker in patients with lung cancer.

**Impact:**

CXCL14 might serve as a potential diagnostic and prognostic biomarker in patients with lung cancer.

## Introduction

Lung cancer is the leading cause of cancer-related deaths worldwide, with 2.21 million new cases and 1.80 million deaths in 2020 (1). At present, the 5-year survival rate of lung cancer patients is only 19%, among which 57% of lung cancer is diagnosed at an advanced stage, and the 5-year survival rate is only 5% (2). As a malignant tumor with high incidence and mortality, early detection of lung cancer is very important. At present, low-dose computed tomography (LDCT) is widely used to screen people with an elevated risk of developing lung cancer and has been shown to effectively reduce lung cancer mortality by at least 20% (3–5). However, the high false positives of LDCT incur unnecessary treatment, increased cost, and negative psychosocial consequences (6–8). Effective biomarkers need to be discovered to refine the existing screening methods and help in making clinical decisions and eventually improve lung cancer outcomes.

Oncogene activation along with persistent inflammation activates transcription factors (NF-κB, STAT3, HIF1α) in tumor cells, resulting in the secretion of chemokines, cytokines, and prostaglandins to recruit inflammatory cells (9). On the one hand, inflammatory cells, such as activated neutrophils and macrophages, are capable of producing reactive oxygen species that bind to and damage DNA in proliferating cells to promote carcinogenesis (10). On the other hand, inflammatory cells consistently produce chemokines and cytokines to gather more of their kin in the tumor microenvironment, exacerbating DNA damage, which may lead to an uncertain clinical outcome (11, 12).

Previous studies have indicated positive associations between abnormal circulating cytokines and the etiology of lung cancer. For instance, an elevation in serum interleukin-6 and interleukin-8 is detected at the time of diagnosis in patients with lung cancer (13). Serum levels of C-reactive protein (CRP), serum amyloid A (SAA), soluble tumor necrosis factor receptor 2 (sTNFRII), and monokines induced by gamma interferon (CXCL9/MIG) are associated with a prospective risk of lung cancer (14). There are also several studies that have investigated the correlation between cytokines and the prognosis of lung cancer. A previous study demonstrated that increased pretreatment serum macrophage colony-stimulating factor (M-CSF) levels indicated poor survival in patients with nonsmall cell lung cancer (15). Allin et al. found that elevated circulating levels of CRP are associated with poor prognosis in several solid cancer types, including lung cancer (16).

Considering the unmet need for reliable lung cancer biomarkers and the relationship between cytokines and the diagnosis and prognosis of lung cancer, we used a multiple Luminex assay to detect abnormal plasma cytokines in patients with lung cancer and assessed the diagnostic and prognostic performance of the selected protein in subsequent research.

## Materials and Methods

### Study Population

Patients with lung cancer and healthy controls who donated blood and/or urine samples were recruited at Tianjin Medical University Cancer Institute and Hospital from August 2015 to December 2017. Patients with pathologically or histologically confirmed lung cancer (prior to any anticancer treatment) and who were not simultaneously diagnosed with other malignant diseases were included in this study. Among all the subjects, 286 patients and 80 healthy controls donated blood samples, and 284 patients and 122 healthy controls donated urine samples; 84 patients donated both blood and urine samples. Human blood and urine samples were collected after an informed consent form was signed.

A total of 133 paraffin-embedded pathological non-small cell lung cancer (NSCLC) and normal lung tissue slides were collected from the Department of Lung Cancer, Tianjin Medical University Cancer Institute and Hospital. Patients with pathologically confirmed nonsmall cell lung cancer undergoing radical surgical resection without preoperative treatment were included. Age, gender, smoking history, cancer stage and death date were collected retrospectively through the medical record registration system.

The Tianjin Medical University Cancer Institute and Hospital ethics committee approved this study (approval number: bc2016014, bc2018009, bc2019091). The study methodologies conformed to the standards set by the Declaration of Helsinki.

### Collection and Preparation of Blood and Urine Samples

Fasting blood was collected in BD Medical System Vacutainer K (2) EDTA plastic tubes and centrifuged at 400 g for 10 minutes at 4°C. Plasma was collected and stored at -80°C for further usage.

Morning urine (20-30 ml) was centrifuged at 5000 g for 40 minutes at 4°C. The urinary supernatants were diluted with phosphate buffer, pH 7.5, and then, the urinary protein was fixed on 22 μm nitrocellulose membranes (Millipore, Bedford, MA) *via* vacuum filtration and stored at -80°C.

For urinary protein extraction, the nitrocellulose membrane was cut into pieces and dissolved in acetone containing 0.5% ammonium bicarbonate with vigorous vortexing. Then, the solution was incubated at 55°C for 60 min and vigorously vortexed every 20 min. Urinary protein was precipitated at 4°C followed by centrifugation at 12000 r/min for 15 min. The protein precipitate was dried at room temperature and redissolved in lysis buffer containing 100 mM Tris, 150 mM NaCl, 1 mM EGTA, 1 mM EDTA, 1% Triton X-100, and 0.5% sodium deoxycholate, and the protein concentration was detected using a Pierce BCA assay (Thermo Fisher Scientific, Waltham, MA, USA). The urinary protein solution was stored at -80°C.

### Screening of Cytokines Using the Multiplex Luminex System

The levels of 20 cytokine molecules were measured using a Luminex-based bead array: FABP4, IL-19, CCL28, CXCL6, CCL20, CCL7, CXCL13, BMP-10, MICB, CCL26, IL-36B, BMP-2, IL-6, IL-1A, IL-1B, GDNF, CXCL9, CXCL14, CCL22 and S100A9. A Magnetic Luminex^®^ Assay (R&D Systems, Minneapolis, Canada) was used following the manufacturer’s instructions for the detection of multiple cytokines in the plasma. Briefly, plasma, standards, and microparticles were incubated in a 96-well plate that was precoated with cytokine-specific antibodies. The immobilized antibodies were able to bind the cytokines of interest after 2 h of incubation. Then, the plate was washed, and incubation with a biotinylated antibody cocktail specific to the cytokines of interest was performed for 1 h. A second wash was performed to remove the unbound biotinylated antibodies. Furthermore, a streptavidin-phycoerythrin conjugate was added to each well to bind to the biotinylated antibodies. After a final wash, the microparticles were resuspended in buffer and assessed using a Luminex^®^ 200™ Analyzer (R&D systems, Minneapolis, MN, Canada).

### Enzyme-Linked Immunosorbent Assay

The CXCL14 level in plasma and urine was assayed using commercially available sandwich enzyme-linked immunosorbent assay kits, Human CXCL14/BRAK DuoSet ELISA and DuoSet ELISA Ancillary Reagent Kit 2(Lot DY866&DY008, R&D Systems). The sensitivity limit of the CXCL14 assay was 31.25 to 4000 pg/ml. Then, 100 µl plasma or 100 µg urinary protein was added to each well. The analyses were performed according to the manufacturer’s instructions.

### Immunohistochemical Staining

FFPE tissue slides were dehydrated and rehydrated. Then, antigen retrieval was carried out by high-pressure heating. The slides were incubated with anti-CXCL14 antibody (1:200, Lot Ab137541; Abcam, Cambridge, UK) overnight at 4°C. After incubation with secondary antibody (Origene, Rockwell, USA) for 1 h at room temperature, the sections were exposed to DAB substrate (Origene, Rockwell, USA) and counterstained with hematoxylin (Solarbio, Beijing, CN).

Slides were scored by two impartial technicians for overall staining intensity and the percentage of cells stained. The proportion scores ranged from 0 to 4 (0, none; 1, 1~25%; 2, 26~50%; 3, 51~75%; and 4, >75%), and the intensity scores ranged from 0 to 3 (0, none; 1, weak; 2, intermediate; and 3, strong). These scores were added to obtain a final score ranging from 0, 2 to 7. Cases scoring 0, 2~4 were considered to have low expression; cases scoring 5~7 were considered to have high expression (17).

### Immunofluorescence Staining of FFPE Slides

FFPE tissue slides were dehydrated and rehydrated. Then, antigen retrieval was carried out by high-pressure heating. The cells were blocked in PBS with 2% BSA and 0.3% Triton for 1 hour with gentle shaking. Then, the slides were incubated with anti-CXCL14 antibody and Anti-pan Cytokeratin antibody (1:200, Lot ab137541 and ab27988; Abcam, Cambridge, UK) overnight at 4°C. After incubation with secondary antibody (Lot 715-585-150 and 711-545-152; Jackson ImmunoResearch, USA) for 2 h at room temperature, mountant with DAPI was added.

### Statistical Analyses

Statistical analysis was performed using SPSS 25.0 (SPSS Inc., Chicago, IL, USA) and GraphPad Prism (GraphPad Software Inc., La Jolla, CA, USA). Receiver operating characteristic (ROC) curves were employed to display the cut-off between sensitivity and specificity for biomarkers able to differentiate between patients and healthy controls. A Mann-Whitney U test was applied to explore differences between different groups. Correlation analysis was performed with Fisher’s exact test. Univariate and multivariate survival analyses were performed using the Cox proportional hazards regression model. Survival curves were obtained using the Kaplan-Meier method. All the tests were two-sided, and differences were considered statistically significant when *P*<0.05.

## Results

### Screening of Elevated Plasma Inflammatory Factors in Patients With Lung Cancer

**Figure 1:**
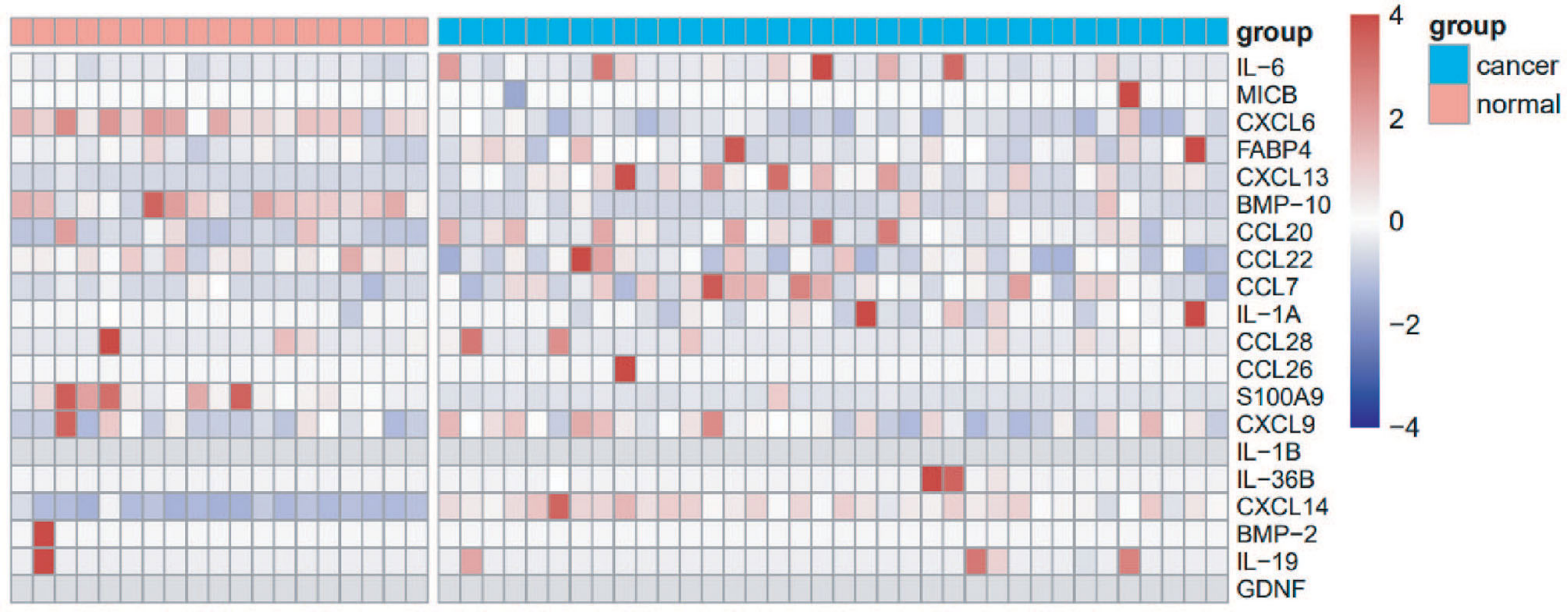
Screening of cytokines using the multiplex Luminex System. Heat map showing the expression level of 20 cytokines in the plasma of 36 patients with lung cancer and 19 healthy controls.

Plasma samples from 19 healthy volunteers and 36 lung cancer patients were detected with a Luminex assay to identify differentially expressed inflammatory factors (**Table S1**). Compared with healthy controls, the levels of CXCL14, CXCL13 and CCL20 were increased in the plasma of patients with lung cancer (**Figure 1**). The AUCs of CXCL14, CXCL13 and CCL20 in the screening group were 0.9956, 0.8392 and 0.7924, respectively (**Figure S1**). Therefore, we preliminarily suggested that CXCL14 could be used as a candidate diagnostic marker for lung cancer.

### The Expression Level of CXCL14 in Plasma and Diagnostic Effect Evaluation

We included 286 patients with lung cancer and 80 control subjects for blood tests, with 184 (64.3%) men and 102 (35.7%) women (**Table S2**). The median age was 60 years (range 25-81 years); 168 were smokers, while 118 people never smoked. A total of 202 adenocarcinomas, 52 squamous cell carcinomas, 13 small cell carcinomas, and 19 other carcinoma types were included. There were 137 patients with stage I lung cancer, 39 patients with stage II, 71 patients with stage III, and 39 patients with stage IV. The staging was based on the eighth edition of the TNM classification for lung cancer.

**Figure 2:**
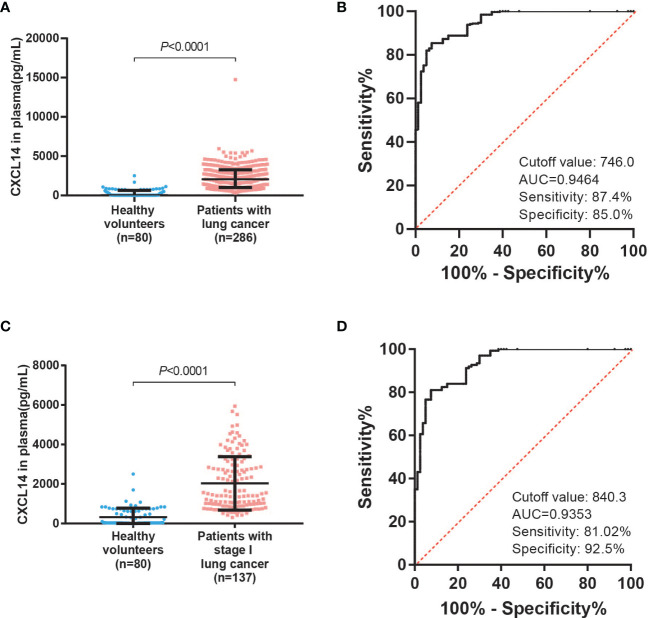
Plasma CXCL14 as a diagnostic biomarker in distinguishing lung cancer patients from control subjects. **(A)** Comparison of the CXCL14 concentration (determined by ELISA) in plasma between control subjects (n=80) and lung cancer patients (n=286) in a retrospective cohort. *P*<0.0001 determined by Mann–Whitney U tests. **(B)** ROC analysis of the diagnostic efficiency of CXCL14 in control subjects versus lung cancer patients in a retrospective cohort (AUC=0.9464, 95% CI: 0.9209–0.9719). **(C)** Comparison of the CXCL14 concentration (determined by ELISA) in plasma between control subjects (n=80) and stage I lung cancer patients (n=137) in a retrospective cohort. *P*<0.0001 determined by Mann–Whitney U tests. **(D)** ROC analysis of the diagnostic efficiency of CXCL14 in control subjects versus stage I lung cancer patients in a retrospective cohort (AUC=0.9353, 95% CI: 0.9034–0.9672). Scatter diagrams present the median values with interquartile ranges.

Plasma samples from lung cancer patients and healthy volunteers were assessed using an ELISA. The median CXCL14 concentration in the plasma of lung cancer patients was 2053.46 pg/ml and that in healthy volunteers was 31.25 pg/ml. Compared with healthy volunteers, plasma CXCL14 levels were 65.7 times higher in lung cancer patients. The plasma CXCL14 level in lung cancer patients was significantly increased (**Figure 2A**, *P*<0.0001). ROC curve analysis showed that plasma CXCL14 achieved an AUC of 0.9464 (95% confidence interval [CI], 0.9209–0.9719) at a cutoff point of 746.0 pg/ml for diagnosis of lung cancer, with 87.4% sensitivity and 85.0% specificity (**Figure 2B**). Further, Plasma level of CXCL14 were also significantly increased in patients with stage I lung cancer (**Figure 2C**, *P*<0.0001). ROC curve analysis showed that plasma CXCL14 achieved an AUC of 0.9353 [95% confidence interval (CI), 0.9034–0.9672] at a cutoff point of 840.3 pg/ml for diagnosis of stage I lung cancer, with 81.02% sensitivity and 92.5% specificity (**Figure 2D**). And we enrolled 57 patients with benign pulmonary nodule and tested the plasma CXCL14 concentration. Histological types included inflammation, hamartoma, tuberculosis, sclerosing pneumocytoma, sclerosing dngioma, fibrotic nodules, liomyoma and alveolar epithelial hyperplasia (**Table S5**). The plasma CXCL14 concentration was slightly higher in patients with lung cancer than in patients with BPNs (Benign pulmonary nodules), but the difference was not significant (cancer vs benign: 2053.46 vs 1611.06 pg/ml, median, P=0.209). The AUC for the plasma CXCL14 concentration to distinguish patients with lung cancer from patients with BPN was 0.5527 (95% CI, 0.477 to 0.6283), with 47.20% sensitivity and 78.95% specificity at the cut-off value of 2253.0 pg/ml (**Figure S3**).

### Correlation Between CXCL14 Expression in the Plasma of Lung Cancer Patients and Clinicopathologic Features

We assessed correlations between the CXCL14 level in plasma and the clinicopathological features of lung cancer patients. There was no significant difference in plasma CXCL14 based on sex (male: 1971.32 pg/ml, female: 2092.14 pg/ml, *P*=0.793), age (≤65 yrs: 2149.06 pg/ml, >65 yrs 1824.33 pg/ml, *P*=0.712), or smoking history (nonsmokers: 1711.00 pg/ml, smokers: 2232.81 pg/ml, *P*=0.411) (**Table S2**). There was a significant difference in plasma CXCL14 level among different histology subtypes (adenocarcinoma: 1647.88 pg/ml, squamous cell carcinoma: 2012.00 pg/ml, SCLC: 2886.00 pg/ml, other malignant types: 2776.21 pg/ml, *P=*0.009). Plasma CXCL14 levels were especially lower in adenocarcinoma than in SCLC (*P*=0.009) and other malignant types (*P*=0.001). Patients with early-stage lung cancer had lower plasma CXCL14 levels than patients with lung cancer at later stages (stage I: 1452.40 pg/ml, stage II: 1898.00 pg/ml, stage III: 2231.00 pg/ml, stage IV: 2661.62 pg/ml, *P*=0.041).

### The Expression Level of CXCL14 in Urine and Diagnostic Effect Evaluation

We also included 284 patients with lung cancer and 122 control subjects for urine tests, with 158 (55.6%) men and 126 (44.4%) women (**Table S3**). The median age was 60 years (range 28-81 years); 136 were smokers, while 124 people never smoked. A total of 219 adenocarcinomas, 47 squamous cell carcinomas, 2 small cell carcinomas, and 16 other carcinoma types were included. There were 158 patients with stage I lung cancer, 48 patients with stage II, 53 patients with stage III, and 25 patients with stage IV. The staging was based on the eighth edition of the TNM classification for lung cancer.

**Figure 3:**
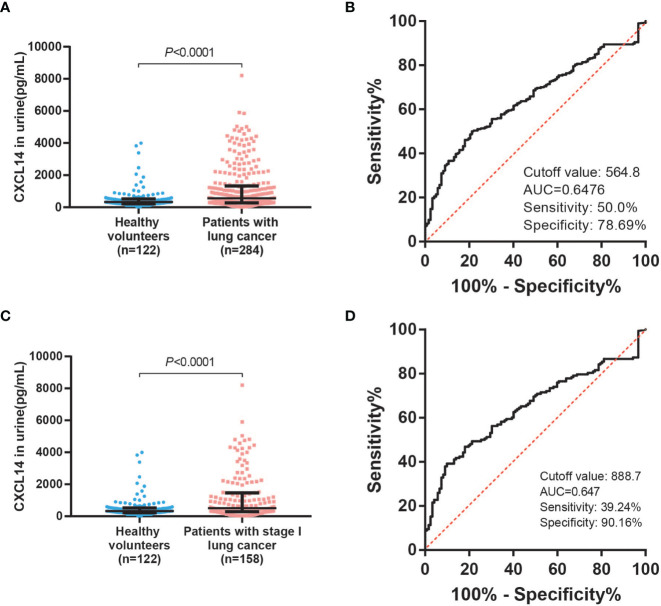
Urinary CXCL14 as a diagnostic biomarker in distinguishing lung cancer patients from control subjects. **(A)** Comparison of the CXCL14 concentration (determined by ELISA) in urine between control subjects (n=122) and lung cancer patients (n=284) in a retrospective cohort. *P*<0.0001 determined by Mann–Whitney U tests. **(B)** ROC analysis of the diagnostic efficiency of urinary CXCL14 in a retrospective cohort of control subjects versus lung cancer patients (AUC=0.6476, 95% CI: 0. 0.5934–0.7091, *P*<0.0001). Scatter diagrams present the median values with interquartile ranges. **(C)** Comparison of the CXCL14 concentration (determined by ELISA) in urine between control subjects (n=122) and stage I lung cancer patients (n=158) in a retrospective cohort. *P*<0.0001 determined by Mann–Whitney U tests. **(D)** ROC analysis of the diagnostic efficiency of CXCL14 in control subjects versus stage I lung cancer patients in a retrospective cohort (AUC=0.647, 95% CI: 0.5829–0.7111). Scatter diagrams present the median values with interquartile ranges.

Urine samples from lung cancer patients and healthy volunteers were assessed using an ELISA. The median CXCL14 concentration in the urine of lung cancer patients was 561.13 pg/ml and that in healthy volunteers was 326.16 pg/ml. Compared with healthy volunteers, urine CXCL14 levels were 1.7 times higher in lung cancer patients. The urine CXCL14 level in lung cancer patients was increased (**Figure 3A**, *P*<0.0001). ROC curve analysis showed that urine CXCL14 achieved an AUC of 0.6476 (95% confidence interval [CI], 0.5934–0.7091) at a cutoff point of 564.8 pg/ml for diagnosis of lung cancer, with 50.0% sensitivity and 78.69% specificity (**Figure 3B**). Further, urine level of CXCL14 were also increased in patients with stage I lung cancer (**Figure 3C**, *P*<0.0001). ROC curve analysis showed that urine CXCL14 achieved an AUC of 0.647 (95% confidence interval [CI], 0.5829–0.7111) at a cutoff point of 888.7 pg/ml for diagnosis of stage I lung cancer, with 39.24% sensitivity and 90.16% specificity (**Figure 3D**).

### Correlation Between CXCL14 Expression in the Urine of Lung Cancer Patients and Clinicopathologic Features

We assessed correlations between the CXCL14 level in urine and the clinicopathological features of lung cancer patients. There was no significant difference in urine CXCL14 level among different histology subtypes (adenocarcinoma: 516.78 pg/ml, squamous cell carcinoma: 569.22 pg/ml, SCLC: 1624.59 pg/ml, other malignant types: 580.77 pg/ml, *P=*0.691), smoking history (nonsmokers: 627.68 pg/ml, smokers: 485.40 pg/ml, *P*=0.574) (**Table S3**), or urine CXCL14 level among different stages (stage I: 506.23 pg/ml, stage II: 629.87 pg/ml, stage III: 569.22 pg/ml, stage IV: 346.12 pg/ml, *P*=0.389). There was a significant difference in urine CXCL14 based on sex (male: 474.09 pg/ml, female: 602.98 pg/ml, *P*=0.048), age (≤65 yrs: 463.54 pg/ml, >65 yrs 769.05 pg/ml, *P*=0.049). In addition, 84 patients donated both blood and urine samples. Then we conducted correlation analysis on CXCL14 levels between blood and urine samples. The results proved that there was no significant correlation between them (**Figure S2**, P=0.661).

### CXCL14 is Differentially Expressed in the Stromal and Tumor Compartments of Lung Cancer

**Figure 4:**
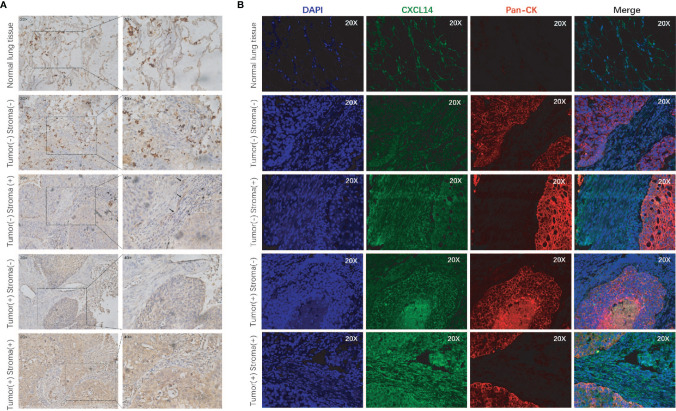
CXCL14 expression in lung cancer tissues. **(A)** Representative images of immunohistochemical staining of CXCL14 in normal lung tissue and lung cancer tissues; magnification, ×20 (left) and ×40 (right). **(B)** Representative images of immunofluorescence staining of CXCL14 in normal lung tissue and lung cancer tissues. The left column shows the cell nuclei in blue (DAPI); the next column shows the presence of CXCL14 in green; the third column shows the cancer cells in red (pancytokeratin), and the final column shows a merged image of the three channels.

We included 94 males (71%) and 39 women (29%) in this analysis (**Table 1**). The average age was 59.4 years (range 40-82 years). There were 27 patients in stage I, 29 in stage II, and 77 patients in stage III. Immunohistochemical staining and immunofluorescence staining showed that CXCL14 was mainly expressed in the cytoplasm (**Figures 4A, B**). The percentages of high CXCL14 expression in stromal fibroblasts and cancer cells were 57% (69/121) and 29.3% (39/133), respectively. Among these patients, 25 showed high expression in both compartments, 44 showed high expression in only in stromal fibroblasts, and 14 showed high expression only in cancer cells. These data suggest that CXCL14 is differentially distributed in stromal fibroblasts and cancer cells.

**Table 1:** Correlation between CXCL14 expression in tissue and clinicopathological characteristics of lung cancer patients.

CXCL14 Expression						
IHC Score	Stroma tissue			Tumor tissue		
	Low Expression (n=52)	High Expression (n=69)	P	Low Expression (n=94)	High Expression (n=39)	P
Age						
Median (range)	60 (41-82)	58 (40-77)	0.500	58 (40-77)	61 (49-82)	0.855
Gender						
Male	40	45	0.354	66	28	0.855
Female	12	24		28	11	
Smoking history						
Yes	41	43	0.147	65	27	0.993
No	11	26		29	12	
Histology						
LUAD	20	27	0.546	35	18	0.262
LUSC	31	37		56	18	
LUASC	1	5		3	3	
Pathologic stage						
I	13	9	**<0.001**	14	13	0.104
II	27	4		23	6	
III	22	56		57	20	
pT stage						
T1	10	15	0.319	19	9	0.371
T2	19	16		24	14	
T3	15	33		38	14	
T4	8	5		13	2	
pN stage						
N0	26	11	**<0.001**	26	14	0.638
N1	12	4		13	5	
N2	14	54		55	20	
Recurrence and metastasis						
Yes	18	38	**0.014**	49	16	0.244
No	34	31		45	23	

Bold text means differences were considered statistically significant when P < 0.05.

### Associations of CXCL14 Expression in Tissue With Clinicopathologic Characteristics

To explore the clinical relevance of CXCL14 in lung cancer, associations between clinicopathological characteristics and epithelial or stromal CXCL14 expression were analyzed (**Table 1**). Stromal CXCL14 expression was significantly associated with advanced pathologic stage (*P*<0.001), pathologic N stage (*P*<0.001), and recurrence and metastasis (*P*=0.014). However, cancer cell CXCL14 expression was not correlated with any clinicopathological characteristics of the patients.

### Associations of CXCL14 Expression in Tissue With PFS and OS

**Figure 5:**
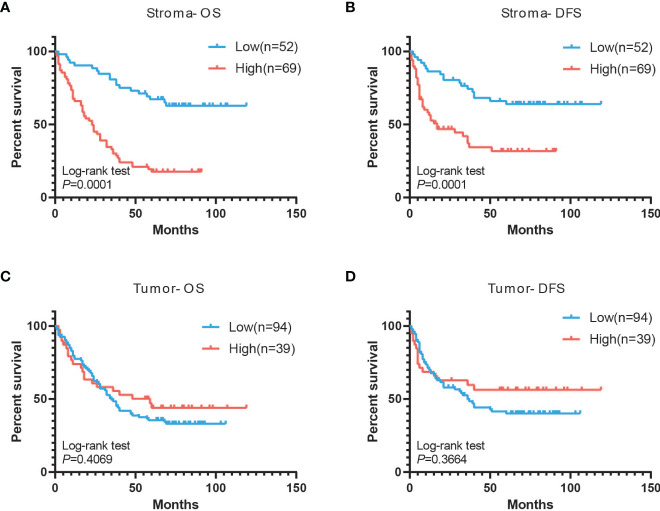
Associations of CXCL14 expression in tissue with PFS and OS. **(A, B)** Kaplan-Meier analysis of overall survival and disease-free survival of lung cancer patients with stromal CXCL14 expression. **(C, D)** Kaplan-Meier analysis of overall survival and disease-free survival of lung cancer patients with tumor CXCL14 expression.

The mean follow-up time for these patients was 43.5 months (range 1–119 months). Kaplan–Meier plots showed that the 10-year OS rates and the 10-year DFS rates were 37.6% and 51.1% of all patients, respectively. The 10-year OS rates for patients with low and high CXCL14 expression in stromal fibroblasts were 63.5% and 20.3% (χ^2^ =23.07, *P* <0.001), respectively, and the 10-year DFS rates were 65.4% and 44.9% (χ^2^ =4.95, *P* =0.026), respectively (**Figures 5A, B**). However, no significant association was observed between CXCL14 expression in cancer cells and 10-year DFS (χ^2^ =1.35, *P* =0.245) or 10-year OS (χ^2^ =1.71, *P* =0.191) (**Figures 5C, D**). Multivariate analysis using the Cox proportional hazard model showed that variables associated with OS and PFS included pathologic N stage, recurrence and metastasis, and CXCL14 expression level in stromal fibroblasts (**Table S4**).

## Discussion

Chemokines are small signaling proteins (8-10 kDa) produced by all cell types in the body, which function by inducing directional chemotaxis of nearby reactive cells and regulating homeostasis and inflammation of the local environment (18). By recruiting different immune cells into the tumor microenvironment, chemokines can directly and indirectly affect cancer progression and patient outcomes (19). CXCL14 is one of the chemokines whose function is quite obscure. It was initially identified as a highly expressed chemokine in breast and kidney tissues (BRAK) and is a member of the CXC chemokine family located on human chromosome 5 q31 (20). The effect of CXCL14 on tumor growth depends on the cell type expressing the factor. In colorectal, head and neck cancers and hepatocellular cell carcinoma, CXCL14 suppresses tumor progression and is correlated with better survival. However, in pancreatic and breast cancer, high stromal CXCL14 expression is associated with increased invasiveness, leading to poor survival in patients (21).

In our study, we used plasma samples to further confirm CXCL14 expression in a cohort of lung cancer patients. We discovered significantly elevated CXCL14 levels in the plasma of patients with lung cancer. Plasma CXCL14 predicts the diagnosis of lung cancer with an AUC of 0.9464 at a cutoff point of 746.0 pg/ml. This cutoff point provides 87.4% sensitivity and 85.0% specificity, which indicates that plasma CXCL14 can be used as a potential diagnostic marker for lung cancer. But the plasma CXCL14 concentration was slightly higher in patients with lung cancer than in patients with BPNs (Benign pulmonary nodules), and the difference was not significant (cancer vs benign: 2053.46 vs 1611.06 pg/ml, P=0.209). It will take a long time to enroll patients with benign pulmonary diseases in a cancer hospital. And the small sample of benign patients may lead to deviance of the above results. In the future, we will recruit more benign patients to optimize our study.

Interestingly, we also tested urine samples from 284 patients with lung cancer and 122 control subjects. The median CXCL14 concentration in the urine of lung cancer patients was 561.13 pg/ml and that in healthy volunteers was 326.16 pg/ml. Compared with healthy volunteers, urinary CXCL14 levels were increased 1.7 times. The urinary CXCL14 level in lung cancer patients was higher than that in healthy volunteers. ROC curve analysis showed that urinary CXCL14 predicted the diagnosis of lung cancer with an AUC of 0.6476 (95% confidence interval [CI], 0.5934–0.7091) at a cutoff point of 564.8 pg/ml. This cutoff point provided 50.0% sensitivity and 78.69% specificity. Urinary CXCL14 levels were correlated with sex (male: 474.09 pg/ml, female: 602.98 pg/ml, *P*=0.048) and age (≤65 yrs: 463.54 pg/ml, >65 yrs 769.05 pg/ml, *P*=0.049). Consistent with the results in plasma, CXCL14 levels were also higher in the urine of lung cancer patients than in the control group. But we conducted correlation analysis on CXCL14 levels between blood and urine samples. The results proved that there was no significant correlation between them(P=0.661).

Based on the above results, we assume that when a tumor develops in the body, CXCL14 is secreted into the blood to play a corresponding role, resulting in increased tumor-related chemokine levels in peripheral blood. Urine is produced by glomerular filtration of blood flowing through the kidney. Blood with an increased CXCL14 level results in urine with the same chemokine increase. Unfortunately, our results do not support this assumption, further sample capacities may be needed to test this hypothesis. However, no studies have been conducted to demonstrate the level of CXCL14 and its clinical role in a large cohort of urine samples from patients with lung cancer.

Although much effort has been made to search for specific cancer biomarkers, few have successfully been adapted for clinical applications. Clinical validation of an autoantibody panel test for lung cancer demonstrated a sensitivity and specificity of 36-39% and 89-91% respectively in three cohorts (22). A previous test for pan-cancer early detection combined NGS analysis of ctDNA in blood with a large panel of protein biomarkers and showed a specificity of 99% and sensitivity of 59% for lung cancer in 104 patients (23). Our study suggests that the plasma CXCL14 level may be a potential marker to assist lung cancer diagnosis with relatively satisfying sensitivity and specificity.

Using IHC, CXCL14 expression was evaluated in both tumor and stromal tissues. CXCL14 expression in tumor tissues was not significantly correlated with histological type, age, sex or pathological stage. However, CXCL14 expression in stromal tissues was significantly correlated with pathological stage, pN stage, recurrence and metastasis. In addition, CXCL14 is an independent prognostic biomarker. Patients with higher stromal CXCL14 had shorter PFS (P<0. 001) and OS (P<0. 001). These results are consistent with the latest findings reported by Xiaoqin Ji et al, who also analyzed CXCL14 expression in lung cancer tumors and stromal tissue and found a significant correlation between CXCL14 expression and pathological staging in stromal tissue only (24). Similarly, in a study by Elin Sjoberg et al., the expression of CXCL14 in breast cancer tissue and tumor tissue was analyzed, and only the expression of CXCL14 in the stroma was related to pathological stage and was an independent marker of breast cancer-specific and recurrence-free survival (25). They also discovered that CXCL14 secreted by surrounding fibroblasts increases the expression of mesenchymal markers and induces epithelial‐mesenchymal transition (EMT) and metastasis (26). In prostate cancer, CXCL14-producing fibroblasts can also enhance proliferation and migration *in vitro* and angiogenesis *in vivo (* 27). Therefore, we assumed that increased CXCL14 secretion by fibroblasts in the microenvironment promoted tumor metastasis, resulting in poor prognosis. Meanwhile, in lung cancer cells, CXCL14 is usually perceived to be downregulated (28). Re-expression or overexpression of CXCL14 in lung cancer cells can suppress tumor growth *in vivo* in an autocrine or paracrine manner (29, 30). CXCL14 may have specific functions in different cell types.

This study has some limitations. First, the sources of blood and urine samples were not the same patients, which may result in a certain degree of bias. Second, the demographic characteristics of the healthy controls did not completely match those of the lung cancer patients. The age range of healthy controls was too wide and included more elderly people. Third, we used a retrospective cohort to confirm the diagnostic effect of CXCL14, which is not enough for a single biomarker. A larger, prospective validation still needs to be conducted. Additionally, the mechanism of high stromal CXCL14 as a negative prognostic factor remains to be investigated.

Overall, the level of CXCL14 in plasma can be used as a marker for early diagnosis of lung cancer at a cutoff point of 746.0 pg/ml. And the level of CXCL14 in urine can be used as a marker for early diagnosis of lung cancer at a cutoff point of 564.8 pg/ml. High expression of CXCL14 in stroma may be a prognostic factor for lung cancer in predicting poor overall survival and disease-free survival. This protein has great potential as a diagnostic and prognostic marker in lung cancer.

## Data Availability Statement

The raw data supporting the conclusions of this article will be made available by the authors, without undue reservation.

## Ethics Statement

The studies involving human participants were reviewed and approved by Tianjin Medical University Cancer Institute and Hospital ethics committee. Written informed consent for participation was not required for this study in accordancewith the national legislation and the institutional requirements.

## Author Contributions

P-FT, Y-CM, D-SY, FL performed writing original draft and conceptualization; specifically performing the experiments. G-ZW, BZ, C-LW performed writing –review and editing; conceptualization; Supervision. C-GL, CC, HZ, X-YS, W-HH, Z-FZ, G-BZ performed data collection.

## Funding

This work was supported by National Key Research and Development Program of China Grant (grant number 2016YFC0905501 to C-LW); National Natural Science Foundation of China (grant number 81772484 to C-LW).

## Conflict of Interest

The authors declare that the research was conducted in the absence of any commercial or financial relationships that could be construed as a potential conflict of interest.

## Publisher’s Note

All claims expressed in this article are solely those of the authors and do not necessarily represent those of their affiliated organizations, or those of the publisher, the editors and the reviewers. Any product that may be evaluated in this article, or claim that may be made by its manufacturer, is not guaranteed or endorsed by the publisher.

## Supplementary Material

Supplementary Figure 1 **(A, B)** Comparison of the CXCL14 concentration (determined by Luminex) in plasma between control subjects (n=19) and lung cancer patients (n=36) in a retrospective cohort. P<0.0001 determined by Mann– Whitney U tests. ROC analysis of the diagnostic efficiency of CXCL14 in control subjects versus lung cancer patients in a retrospective cohort (AUC=0.9956, 95% CI: 0.9849–1.006). Scatter diagrams present the median values with interquartile ranges. **(C, D)** Comparison of the CXCL13 concentration (determined by Luminex) in plasma between control subjects (n=19) and lung cancer patients (n=36) in a retrospective cohort. P<0.0001 determined by Mann–Whitney U tests. ROC analysis of the diagnostic efficiency of CXCL13 in control subjects versus lung cancer patients in a retrospective cohort (AUC=0.8392, 95% CI: 0.7358–0.9426). Scatter diagrams present the median values with interquartile ranges. **(E, F)** Comparison of the CCL20 concentration (determined by Luminex) in plasma between control subjects (n=19) and lung cancer patients (n=36) in a retrospective cohort. P<0.0001 determined by Mann–Whitney U tests. ROC analysis of the diagnostic efficiency of CCL20 in control subjects versus lung cancer patients in a retrospective cohort (AUC=0.7924, 95% CI: 0.645–0.9398). Scatter diagrams present the median values with interquartile ranges.

Supplementary Figure 2 Correlation analysis of CXCL14 in plasma and urine. Pearson correlation analysis of CXCL14 in plasma and urine in 84 patients (P=0.661).

Supplementary Figure 3 **(A)** Comparison of the CXCL14 concentration (determined by ELISA) in plasma between benign patients (n=57) and lung cancer patients (n=286) in a retrospective cohort. P=0.209 determined by Mann–Whitney U tests. **(B)** ROC analysis of the diagnostic efficiency of CXCL14 in benign patients versus lung cancer patients in a retrospective cohort (AUC=0.5527, 95% CI: 0.477 to 0.6283).

## Abbreviations

CXCL14, CXC chemokine ligand 14; ELISA, enzyme-linked immunosorbent assay; IHC, immunohistochemical; IF, immunofluorescence; FFPE, formalin fixed paraffin-embedded; AUC, area under the curve; ROC, receiver operating characteristic; LDCT, low-dose computed tomography.
