# Supplementary figures and images for: Plasma CXCL14 as a Candidate Biomarker for the Diagnosis of Lung Cancer
Source: Front Oncol. 2022 Jun 8;12:833866. doi: 10.3389/fonc.2022.833866 (PMC9235466; doi:10.3389/fonc.2022.833866)

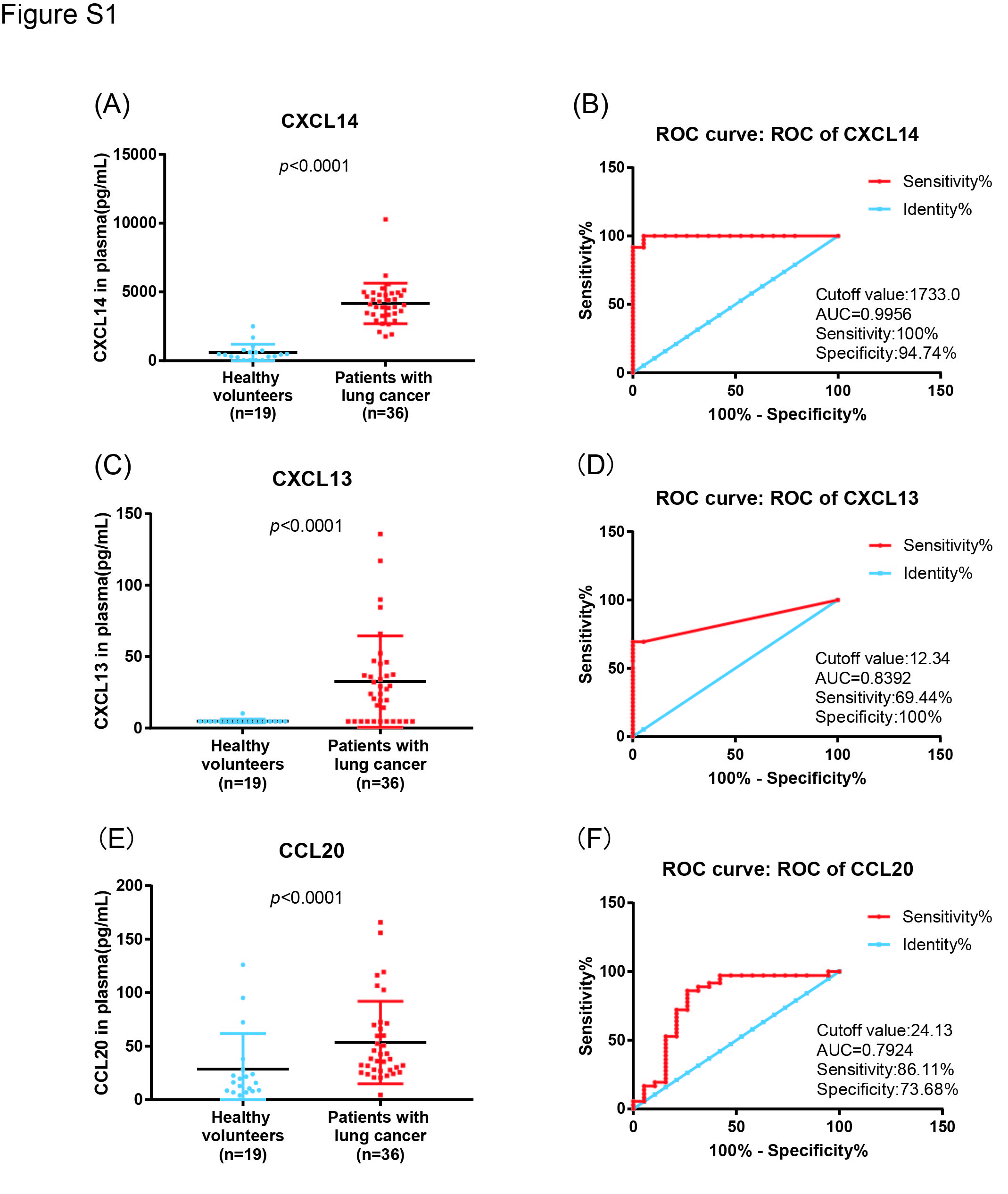

Supplement: Supplementary Figure 1 — (A, B) Comparison of the CXCL14 concentration (determined by Luminex) in plasma between control subjects (n=19) and lung cancer patients (n=36) in a retrospective cohort. P<0.0001 determined by Mann– Whitney U tests. ROC analysis of the diagnostic efficiency of CXCL14 in control subjects versus lung cancer patients in a retrospective cohort (AUC=0.9956, 95% CI: 0.9849–1.006). Scatter diagrams present the median values with interquartile ranges. (C, D) Comparison of the CXCL13 concentration (determined by Luminex) in plasma between control subjects (n=19) and lung cancer patients (n=36) in a retrospective cohort. P<0.0001 determined by Mann–Whitney U tests. ROC analysis of the diagnostic efficiency of CXCL13 in control subjects versus lung cancer patients in a retrospective cohort (AUC=0.8392, 95% CI: 0.7358–0.9426). Scatter diagrams present the median values with interquartile ranges. (E, F) Comparison of the CCL20 concentration (determined by Luminex) in plasma between control subjects (n=19) and lung cancer patients (n=36) in a retrospective cohort. P<0.0001 determined by Mann–Whitney U tests. ROC analysis of the diagnostic efficiency of CCL20 in control subjects versus lung cancer patients in a retrospective cohort (AUC=0.7924, 95% CI: 0.645–0.9398). Scatter diagrams present the median values with interquartile ranges. [file Image_1.jpeg]

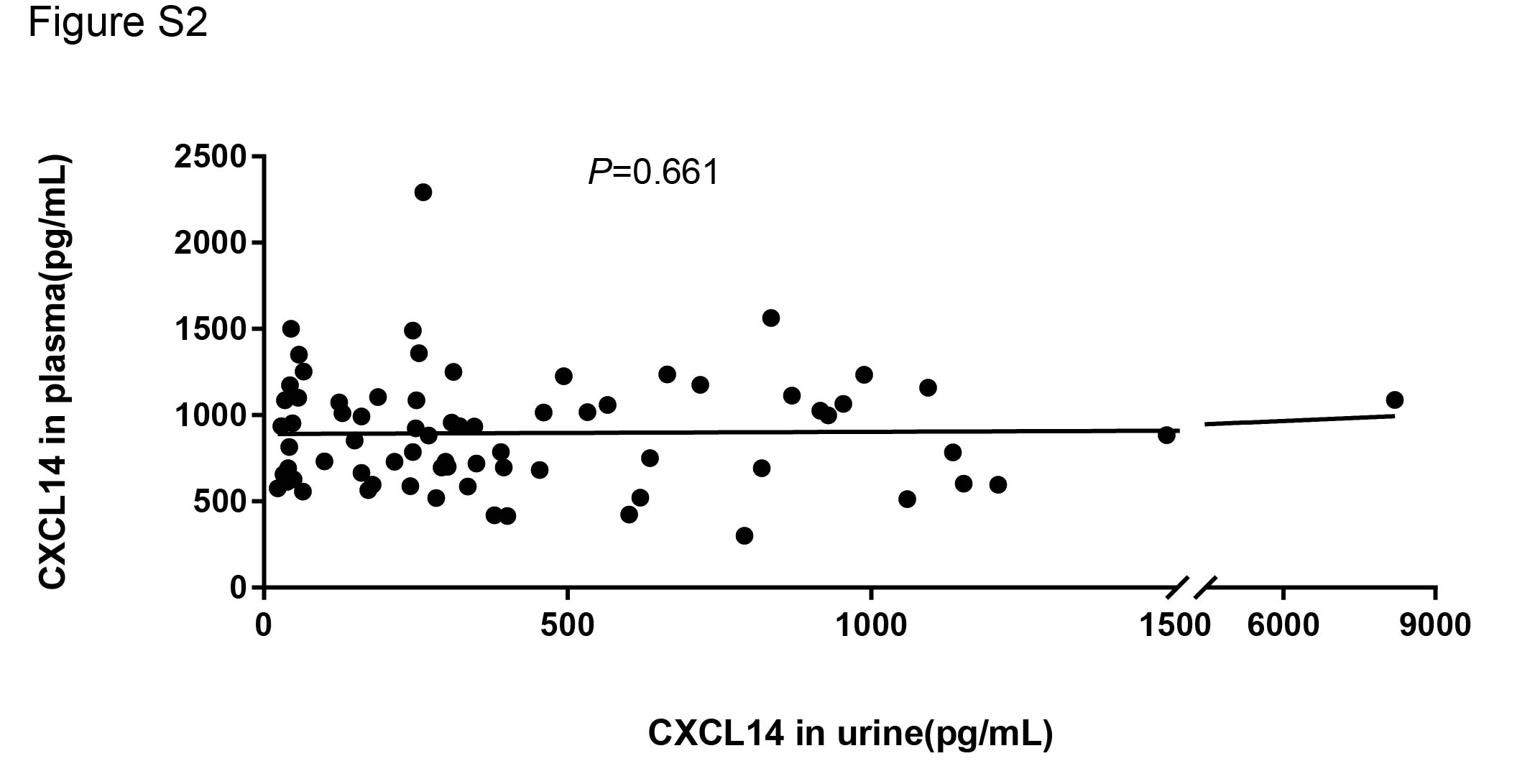

Supplement: Supplementary Figure 2 — Correlation analysis of CXCL14 in plasma and urine. Pearson correlation analysis of CXCL14 in plasma and urine in 84 patients (P=0.661). [file Image_2.jpeg]

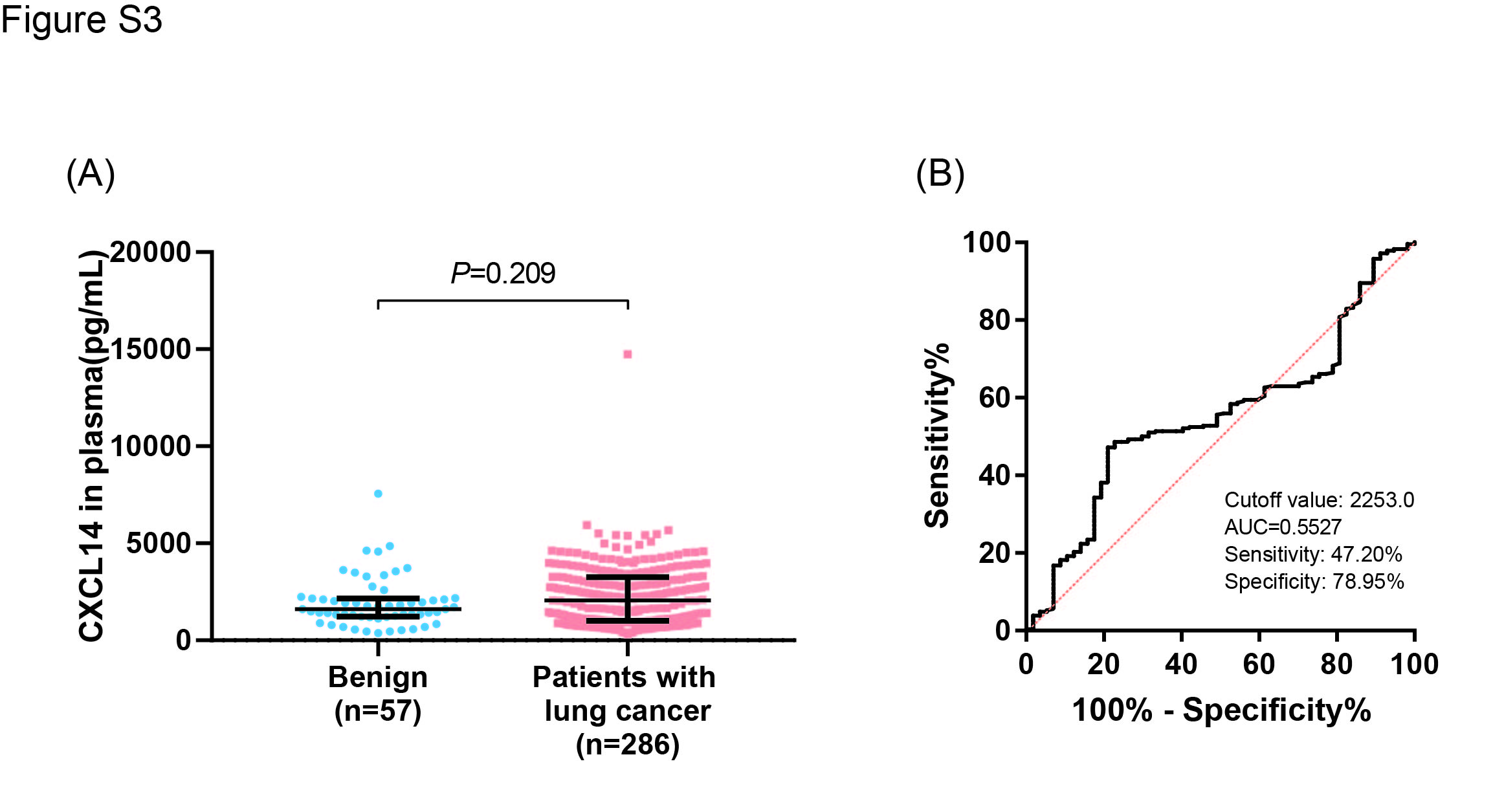

Supplement: Supplementary Figure 3 — (A) Comparison of the CXCL14 concentration (determined by ELISA) in plasma between benign patients (n=57) and lung cancer patients (n=286) in a retrospective cohort. P=0.209 determined by Mann–Whitney U tests. (B) ROC analysis of the diagnostic efficiency of CXCL14 in benign patients versus lung cancer patients in a retrospective cohort (AUC=0.5527, 95% CI: 0.477 to 0.6283). [file Image_3.jpeg]
