# Supplementary material for: Plasma CXCL14 as a Candidate Biomarker for the Diagnosis of Lung Cancer
Source: Front Oncol. 2022 Jun 8;12:833866. doi: 10.3389/fonc.2022.833866 (PMC9235466; doi:10.3389/fonc.2022.833866)
Supplement: Supplementary file 4 [file Table_1.docx]

Table S1 Clinicopathological characteristics of enrolled subjects

| **Variables** | **Plasma** | | | |
| --- | --- | --- | --- | --- |
|  | **n** | **CXCL14 Conc.**  **(pg/ml) (Median)** | **Q1-Q3** | **P value** |
| Healthy controls |  |  |  |  |
| Gender |  |  |  |  |
| Male | 10 | 463.72 | 297.41-586.72 | 0.99 |
| Female | 9 | 331.84 | 306.16-743.07 |  |
| Age |  |  |  |  |
| Median, 32yrs (range, 23–45) | 19 | 452.39 | 280.18-680.07 |  |
| Patients |  |  |  |  |
| Gender |  |  |  |  |
| Male | 21 | 4443.44 | 3344.75-4952.84 | 0.34 |
| Female | 15 | 3894.07 | 3409.06-4387.14 |  |
| Age |  |  |  |  |
| ≤65 | 24 | 4207.02 | 3326.79-4801.47 | 0.99 |
| >65 | 12 | 3757.78 | 3428.83-4838.15 |  |
| Smoking History |  |  |  |  |
| Yes | 24 | 4254.87 | 3326.79-4827.43 | 0.99 |
| No | 12 | 3982.13 | 3441.40-4810.39 |  |
| Unknown |  |  |  |  |
| Pathological Types |  |  |  | 0.575 |
| Adenocarcinoma | 14 | 3898.56 | 3452.79-4678.65 |  |
| Squamous cell carcinoma | 11 | 3837.89 | 2797.43-4854.93 |  |
| Small cell lung cancer | 9 | 4105.19 | 3901.25-4765.32 |  |
| Other malignant types | 2 | 4678.69 | 4541.62-4815.77 |  |
| TNM Stage |  |  |  |  |
| I | 12 | 3898.56 | 3332.78-4552.98 | 0.058 |
| II | 3 | 3272.90 | 2522.18-3858.17 |  |
| III | 13 | 4747.99 | 4061.22-5133.94 |  |
| IV | 8 | 3396.68 | 2681.07-4494.75 |  |

Table S2 Clinicopathological characteristics of enrolled subjects

| **Variables** | **Plasma** | | | |
| --- | --- | --- | --- | --- |
|  | **n** | **CXCL14 Conc.**  **(pg/ml) (Median)** | **Q1-Q3** | **P value** |
| Healthy controls |  |  |  |  |
| Gender |  |  |  |  |
| Male | 55 | 254.20 | 28.57-743.51 | **0.043** |
| Female | 25 | 31.25 | 31.25-31.25 |  |
| Age |  |  |  |  |
| Median, 84yrs (range, 24–99) | 80 | 31.25 | 31.25-632.98 |  |
| Patients |  |  |  |  |
| Gender |  |  |  |  |
| Male | 184 | 1971.32 | 1045.71-3238.0 | 0.793 |
| Female | 102 | 2092.14 | 1002.41-3227.50 |  |
| Age |  |  |  |  |
| ≤65 | 212 | 2149.06 | 973.08-3290.30 | 0.712 |
| >65 | 74 | 1824.33 | 1090.87-2891.25 |  |
| Smoking History |  |  |  |  |
| Yes | 168 | 2232.81 | 1061.85-3290.25 | 0.411 |
| No | 118 | 1711.00 | 993.82-3142.12 |  |
| Unknown | - | - |  |  |
| Pathological Types |  |  |  |  |
| Adenocarcinoma | 202 | 1647.88 | 955.00-2927.87 | **0.009** |
| Squamous cell carcinoma | 52 | 2012.00 | 1023.63-3631.20 |  |
| Small cell lung cancer | 13 | 2886.00 | 1982.65-3417.38 |  |
| Other malignant types | 19 | 2776.21 | 2271.49-3821.67 |  |
| TNM Stage |  |  |  |  |
| I | 137 | 1452.40 | 969.98-2861.00 | **0.041** |
| II | 39 | 1898.00 | 1125.00-3002.50 |  |
| III | 71 | 2231.00 | 985.86-3448.19 |  |
| IV | 39 | 2661.62 | 1789.48-3544.15 |  |
| EGFR |  |  |  |  |
| Wild type | 70 | 2419.93 | 1419.50-3494.67 | 0.610 |
| Mutation | 70 | 2302.85 | 1349.36-3273.10 |  |
| Untested | 146 | 1300.46 | 882.73-3020.18 |  |

Table S3 Clinicopathological characteristics of enrolled subjects

| **Variables** | **Urine** | | | |
| --- | --- | --- | --- | --- |
|  | **n** | **CXCL14 Conc.**  **(pg/ml) (Median)** | **Q1-Q3** | **P value** |
| Healthy controls |  |  |  |  |
| Gender |  |  |  |  |
| Male | 31 | 336.39 | 267.49-392.64 | 0.775 |
| Female | 91 | 307.72 | 173.56-632.06 |  |
| Age |  |  |  |  |
| Median, 35yrs (range, 24–73) | 122 | 326.16 | 191.94-532.22 |  |
| Patients |  |  |  |  |
| Gender |  |  |  |  |
| Male | 158 | 474.09 | 253.3-1147.44 | **0.048** |
| Female | 126 | 602.98 | 329.44-2128.51 |  |
| Age |  |  |  |  |
| ≤65 | 204 | 463.54 | 254.81-1255.57 | **0.049** |
| >65 | 80 | 769.05 | 346.06-1320.86 |  |
| Smoking History |  |  |  |  |
| Yes | 136 | 485.40 | 260.07-2054.83 | 0.574 |
| No | 124 | 627.68 | 267.71-1304.32 |  |
| Unknown | 24 | 433.68 | 333.03-661.51 |  |
| Pathological Types |  |  |  |  |
| Adenocarcinoma | 219 | 516.78 | 254.78-1466.31 | 0.691 |
| Squamous cell carcinoma | 47 | 569.22 | 300.88-826.68 |  |
| Small cell lung cancer | 2 | 1624.59 | 1098.15-2151.04 |  |
| Other malignant types | 16 | 580.77 | 393.77-1049.24 |  |
| TNM Stage |  |  |  |  |
| I | 158 | 506.23 | 284.95-1446.35 | 0.389 |
| II | 48 | 629.87 | 321.30-1590-08 |  |
| III | 53 | 569.22 | 288.86-1151.23 |  |
| IV | 25 | 346.12 | 244.89-651.86 |  |

Table S4 Multivariate analysis of the CXCL14 expression in stroma tissue in overall survival and disease-free survival

| Predictor | OS | | DFS | |
| --- | --- | --- | --- | --- |
|  | P | HR (95%CI) | P | HR (95%CI) |
| pN stage | 0.000 | 1.956(1.429 to 2.678) | 0.000 | 1.810(1.311 to 2.499) |
| Recurrence and metastasis | 0.000 | 4.205(2.451 to 7.216) | - | - |
| CXCL14 expression | 0.01 | 1.608(1.123 to 2.304) | 0.011 | 1.631(1.121 to 2.374) |

| **Variables** | **Plasma** | | | |
| --- | --- | --- | --- | --- |
|  | **n** | **CXCL14 Conc.**  **(pg/ml) (Median)** | **Q1-Q3** | **P value** |
| Benign patients |  |  |  |  |
| Gender |  |  |  |  |
| Male | 29 | 1964.00 | 1406.00-3360.70 | **0.001** |
| Female | 28 | 1319.00 | 833.13-1751.25 |  |
| Age |  |  |  |  |
| ≤65 | 49 | 1611.00 | 1210.00-2155.00 | 0.505 |
| >65 | 8 | 1782.50 | 1432.25-2232.29 |  |
| Smoking History |  |  |  |  |
| Yes | 28 | 2034.50 | 1391.75-3391.98 | **0.001** |
| No | 29 | 1319.00 | 844.88-1746.00 |  |

Table S5 Clinicopathological characteristics of enrolled subjects
